# Single dose epidural hydromorphone in labour pain: maternal pharmacokinetics and neonatal exposure

**DOI:** 10.1007/s00228-020-02880-6

**Published:** 2020-05-03

**Authors:** Terhi Puhto, Merja Kokki, Henriikka Hakomäki, Michael Spalding, Teemu Gunnar, Seppo Alahuhta, Merja Vakkala

**Affiliations:** 1 Department of Anaesthesiology, Medical Research Center Oulu (MRC Oulu), Oulu University Hospital, PO Box 21, 90029 Oulu, Finland; 2 Department of Anaesthesiology and Intensive Care, Kuopio University Hospital, Kuopio, Finland; 3 School of Pharmacy, University of Eastern Finland, Kuopio, Finland; 4 Forensic Toxicology Unit (THL), The Finnish Institute for Health and Welfare, Helsinki, Finland

**Keywords:** Hydromorphone, Pharmacokinetics, Epidural, Labour, Pain

## Abstract

**Introduction:**

Epidural hydromorphone could be useful in obstetric analgesia as there is a need for a more water-soluble opioid than sufentanil or fentanyl with prolonged analgesic effect. To our knowledge, the pharmacokinetics of epidural hydromorphone has not been evaluated in parturients.

**Materials and methods:**

In this pilot study, seven healthy parturients were given a single epidural dose of hydromorphone for labour pain. One parturient received 1.5 mg, two 0.75 mg and four 0.5 mg of hydromorphone hydrochloride. Dose was decreased due to nausea and pruritus. Hydromorphone’s effect, adverse effects and plasma concentrations were evaluated. Neonatal drug exposure was evaluated by umbilical vein and artery opioid concentration at birth. Neonatal outcomes were assessed using Apgar and the Neurologic Adaptive Capacity Score (NACS).

**Results:**

All patients received additional levobupivacaine doses on parturients’ requests. The first dose was requested at a median of 163 min (range 19–303 min) after hydromorphone administration. A total of 12 opioid related expected adverse events were reported by seven parturients. All newborn outcomes were uneventful. Hydromorphone’s distribution and elimination after single epidural dose seem similar to that reported for non-pregnant subjects after intravenous hydromorphone administration, but further research is required to confirm this observation.

**Conclusions:**

The optimal dose of hydromorphone in labour pain warrants further evaluation.

## INRODUCTION

Labour pain is severe in 80% of parturients [1]. Epidural analgesia with bolus doses of local anaesthetics and opioid analgesics is commonly used for pain relief [2]. Lipid soluble opioids, fentanyl and sufentanil, are preferred as the onset of analgesic action is rapid. However, the short duration of effect necessitates repeated dosing [3]. Thus, in prolonged deliveries, the cumulative dose of opioid may become unnecessarily high and may cumulate into the foetus [4].

Lipid solubility of an opioid is one of the most important attributes for onset and duration of analgesia [5]. Very lipid soluble opioids given intrathecally absorb rapidly through the epidural fat into the circulation [3], but probably partially act on the spinal opioid receptors [6]. Lipophobic opioids proceed easier from epidural space to intrathecal space and remain in the cerebrospinal fluid (CSF) longer than lipophilic opioids [6]. Lipophobic opioids exert an analgesic effect at significantly lower doses after spinal administration than they will after intravenous administration. Lipophobic morphine has been evaluated in labour pain, but its slow onset of action, adverse effects and the possibility of late onset respiratory depression makes it less attractive for spinal administration in labour pain treatment [7].

Hydromorphone (HM) is a semisynthetic, selective μ-opioid receptor agonist that was synthesisedsynthesized in 1922 and introduced for clinical use in 1926. HM is a hydrogenated ketone of morphine and is chemically categorized as a phenanthrene [8]. The safety of HM in epidural use has been evaluated in several clinical trials [9], and it has been proposed that the use of intrathecal morphine can be replaced with HM [10]. HM’s onset of analgesia begins 20 min after epidural dosing and analgesia after a single dose lasts for up to 16–24 h [11–13].

Hydromorphone is extensively metabolized to hydromorphone-3-glucuronide and dihydroisomorphine glucuronide by hepatic UDP-Glucuronosyltransferase-2B7-enzyme (UGT2B7) [8]. Hydromorphone-3-glucuronide is an active metabolite that may cause neuroexcitatory effects; however, this has not been well-documented in humans and is expected to only have clinical relevance in patients with renal impairment. Genetic polymorphisms have been described for UGT2B7 and may play a role in interindividual variability of HM clearance [14]. To our knowledge, the elimination of HM has not been studied in neonates. However, Bouwmeester and associates have reported low formation of morphine-3-glucuronide in neonates during their first days of life, glucuronidation process also mediated by UGT2B7 [15]. This finding suggests immature glucuronidation of HM in the neonate, leading to low elimination of the parent drug. The median protein binding of hydromorphone is low 12% [16]; therefore plasma protein changes that occur during pregnancy may not have so much effect on hydromorphone pharmacokinetics or -dynamics.

Epidural HM could be useful in obstetric analgesia as there is a need for a more water-soluble opioid than sufentanil or fentanyl with prolonged analgesic effect. To our knowledge, the pharmacokinetics of HM has not been evaluated after epidural use in parturients. Thus, we have designed the present study to assess the pharmacokinetics, efficacy and safety of HM in labouring patients as well as its effects on the newborn.

## Materials and methods

The study protocol was approved by the Finnish Research Ethics Board approval (TUKIJA) (Dnro 92/06.00.01/2016) and conducted in accordance with the Declaration of Helsinki. The Finnish Medicines Agency was notified (KL nro 119/2016, 07.10.2016), and the study was recorded in the European Clinical Trials Database (EudraCT no. 2016–000903-10). The study had institutional approval.

Parturients were recruited between February 2017 and February 2018. Inclusion criteria were singleton pregnancy, American Society of Anesthesiologists (ASA) I/II physical status, term pregnancy (gestational weeks between 37 and 41), maternal age 18 years or older, maternal wish to have epidural analgesia and informed consent. Exclusion criteria were no informed consent, foetal growth retardation or placental insufficiency, contraindication to epidural analgesia or to HM, body mass index (BMI) over 35 kg/m^2^, immediate need for pain relief or opioid use during the last 12 h.

Women were asked to participate in early labour when their contraction pain was less than 5 on an 11-point numeric rating scale (NRS, 0 = no pain, 10 = most pain). The parturients were provided oral and written information on the trial protocol, and the parturients gave their written informed consent. When parturients wished to have epidural analgesia, an epidural catheter was placed via the lumbar 2–3 or 3–4 interspace using a paramedial approach and loss of resistance to saline. To ensure epidural catheter placement, a test dose of 10 mL lidocaine 5 mg/mL with epinephrine 10 μg/mL was given, and loss of cold sensation was tested with an alcohol swipe. Dose of HM was chosen based on earlier studies [11–13]. Hydromorphone hydrochloride 1.5 mg (corresponding to 1.33 mg of HM free base) was administered epidurally to the first patient, 0.75 mg (0.665 mg of HM free base) to the next two patients and 0.5 mg (0.443 mg of HM free base) to four successive patients (Palladon**®** 2 mg/mL, preservative free, Mundipharma, Vantaa, Finland) mixed with 10 mL saline. The dose was decreased progressively as the higher doses were associated with protracted nausea. Levobupivacaine 1.25 mg/mL 10 mL was used as a rescue medication if the patient required additional pain relief. Maternal blood pressure, heart rate, peripheral oxygen saturation and foetal heart rate using cardiotocography and uterine contractions were monitored. Cardiotocography recordings were evaluated during labour by an attending gynaecologist who performed further procedures (e.g. intrapartum foetal pH measurement) if necessary.

An intravenous cannula for blood sampling was inserted in an antecubital vein. Venous blood samples were taken at 0, 15, 30 and 45 min, and at 1, 2, 3, 4, 6, 8, 10 and 12 h after the epidural HM dosing or until the mother gave birth. The last maternal blood sample was obtained immediately after birth. Umbilical arterial and venous blood samples were drawn after delivery after the umbilical cord had been clamped to estimate the neonatal drug exposure.

Neonatal outcome was assessed using Apgar scores [17] at 1, 5 and 10 min after birth and the Neurologic and Adaptive Capacity Score (NACS) [18] at 30–60 min after birth. A baby with a score of 35 or more is considered to be a vigorous neonate [18].

### Hydromorphone laboratory analysis

Blood samples were collected into BD Vacutainer® K_2_EDTA tubes (reference #368841/2 ml) and stored at − 21 °C until analysis. HM hydrochloride was purchased from Sigma (St. Louis, MO, USA), and a deuterium-labelled HM-d6 solution (0.1 mg/mL in MeOH) was obtained from Cerilliant (Round Rock, Texas, USA) for analytical reference compounds. All solvents and other reagents of analytical grade were supplied by Merck (Darmstadt, Germany) or VWR International (Fontenay-sous-Bois, France). All HM determinations were performed at the Forensic Toxicology Unit in the National Institute for Health and Welfare, Helsinki, Finland.

In analytical sample preparation, 0.5 mL of 0.5 M Na_2_HPO_4_ buffer (pH 9.8) and 3 mL of n-butyl acetate/ethylacetate (1:1, v/v) including 0.05 μL of HM-d6 (0.1 mg/mL in MeOH) were added to 0.5 mL of whole blood. The contents of the test tubes were rapidly and simultaneously vortex-mixed (30 s) in a multitube vortexer. After centrifugation (2500*g*, 5 min), 60 μL of the supernatant were added to 2.0 mL autosampler vials containing 200 μL inserts. The samples were rapidly vortex-mixed (1 s) and capped for analytical determination.

Blood HM concentrations were determined using an Agilent Technologies (Palo Alto, CA, USA) 1290 II ultra-high-performance liquid chromatography (UHPLC) coupled with a 6495A triple quadrupole mass spectrometer (MS/MS). Electrospray ionization in positive mode (ESI+) with a heated nitrogen gas (98%) was applied for ionization. An in-house developed method was linear from 0.1 to 25 ng/mL of HM, and the lower limit of quantification (LLOQ) of HM was 0.1 ng/mL. Concentrations below 0.1 ng/mL were reported as less than LLOQ. Accuracy (bias), repeatability (within-day precision) and time-different intermediate precision (day-to-day precision) were always below 15% for three different concentration levels (0.2; 1; 10 ng/mL) at the validation experiments (three replicates each day for each concentration × 5 days).

No interfering compounds or any selectivity problems were observed. Standard calibration curves of HM were prepared for each analysis batch, and HM-d6 was used in all analyses and daily calibration as an internal standard.

### Hydromorphone pharmacokinetic analysis

The results are expressed as HM free base. Pharmacokinetic parameters were calculated based on a noncompartmental analysis method, using Phoenix WinNonlin software version 6.3 (Certara, Princeton, NJ, USA). The elimination rate constant (kel) was determined from the terminal log-linear phase using 4–7 data points. The terminal half-life (t½) was calculated as ln(2)/kel. Other determined pharmacokinetic parameters were the maximum observed plasma concentration (Cmax) and time to maximum observed plasma concentration (Tmax), area under the concentration-time curve from time 0 to the last quantifiable concentration using the linear trapezoidal rule (AUClast), area under the curve from time 0 extrapolated to infinity (AUCinf), the percentage of the extrapolated area (AUCextrap), the apparent volume of distribution at the terminal phase (Vz/F) and the apparent total body clearance (CL/F). Because three different doses were used, the pharmacokinetic parameters were normalized by the dose for Cmax and the AUCs.

Pharmacokinetic simulation of epidural hydromorphone administration was performed using pharmacokinetic parameters (apparent volume of the central compartment Vc 26.2 L, elimination rate constant from the central compartment k10 0.071 min^−1^, and intercompartmental transfer rate constants k12 0.260 min^−1^, k21 0.110 min^−1^, k13 0.153 min^−1^, k31 0.017 min^−1^) from a previous i.v. study [17] (dose 10 μg/kg, mean dose of 0.73 mg in the study population), and additionally adjusting the absorption rate constant (k_a_) of epidural hydromorphone to 0.015 min^−1^ to get the observed Tmax, and assuming that the absolute bioavailability was 100%. Simulated doses 0.50 mg and 0.75 mg were chosen based on doses used in the present study. Simulation was conducted with delta time of 2 min and Runge-Kutta 4th order algorithm using STELLA Professional software (version 1.1, isee systems Inc. NH, USA).

### Statistical analysis

No formal sample size calculation was performed, but a group of seven parturients was considered to provide sufficient pharmacokinetic data for this pilot study of epidural HM.

The results are presented as median, minimum and maximum or number of parturients as appropriate. The ratios of venous versus arterial umbilical cord drug concentrations were calculated. The Wilcoxon signed-rank test was used to determine whether the ratio was significantly different from unity.

## Results

Seven women aged 22–34 years, agreed to participate. Data regarding the parturients’ demographics, as well as pregnancy- and labour-related details are presented in Table 1.

**Table 1:** Parturients’ age, weight and height, pregnancy- and labour-related details

ID	Age (years)	Weight (kg)	Height (cm)	Gestational age (weeks)	Duration of the first stage (hh:min)	Duration of the second stage (min)	Vaginal delivery/caesarian section
**1**	22	63	160	41	11:33	5	Yes/no
**2**	31	94	168	40	–	–	No/yes
**3**	29	82	165	42	5:50	11	Yes/no
**4**	23	69	164	40	14:10	17	Yes/no
**5**	33	85	166	37	9:45	20	Yes/no
**6**	24	68	167	39	22:00	56	Yes/no
**7**	28	82	173	39	21:10	28	Yes/no

ID, Identification number of the patient; hh:min, hours and minutes

Definition of the 1st stage: cervix attains full dilation and the presenting

foetal part descends

Definition of the 2nd stage: active pushing

There was one major protocol deviation during the study. Due to protracted nausea and vomiting, the dose of HM was eventually decreased from 1.5 to 0.5 mg. A few minor deviations regarding the planned sampling times were also noted. As the actual sampling times were used in the pharmacokinetic calculations, it was considered unlikely that these deviations affected the results. One parturient who had received a dose of 0.75 mg epidurally did not give birth during the 730 min follow-up, and caesarean section due to failure of labour progress and chorioamnionitis was performed 24 h and 55 min after HM administration (Table 1, patient ID 2).

**Fig. 1:**
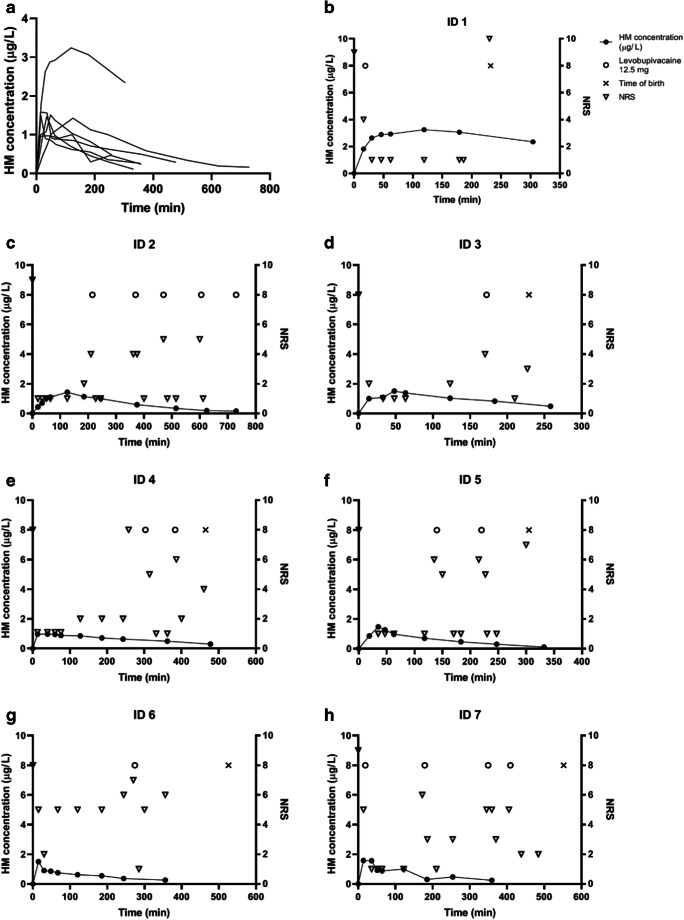
**a** Hydromorphone (HM) plasma concentrations from seven parturients plotted against time after epidural administration of 0.5–1.5 mg of hydromorphone. **b–h** Individual hydromorphone plasma concentrations, NRSs (numeric rating scale for pain), times of birth and levobupivacaine administrations plotted against time from study participants ID 1–7

**Fig. 2:**
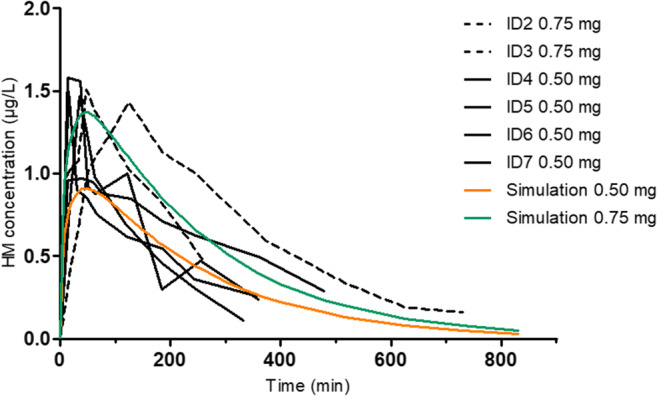
Individual hydromorphone (HM) plasma concentrations from ID 2–7 after epidural administration of 0.5–0.75 mg of hydromorphone, plotted with a simulation curve using distribution and elimination kinetics from Hill et al. (1991) i.v. hydromorphone study conducted with healthy male subjects

HM maternal plasma concentrations are presented in Fig. 1 and pharmacokinetic parameters expressed for HM free base in Table 2. ID1 showed high HM concentrations throughout the sampling period, thus accurate AUCs, T½, Vz/f and CL/F estimates could not be calculated for this individual. As three different doses were used over the course of the study, a dose-normalized Cmax and AUCs were calculated for interindividual comparison. Dose-normalized (per mg of HM free base) median Cmax was 2.44 μg/L/mg (range 2.15–3.57 μg/L/mg), AUClast 487 min μg/L/mg (range 369–713 min μg/L/mg) and AUCinf 619 min μg/L/mg (range 453–938 min μg/L/mg). The relative standard deviations of dose-normalized Cmax, AUClast and AUCinf were 23%, 27% and 28%, respectively. The plasma concentration curve of epidural HM (bioavailability 100%) was simulated using distribution and elimination kinetics from a previously published i.v. study [19], and the simulated curves for doses 0.50 mg and 0.75 mg showed similarity to the observed curves in the present study in parturients (Fig. 2).

**Table 2:** Pharmacokinetic parameters from seven parturients after epidural hydromorphone administration analysed by noncompartmental analysis method

ID	HM dose (mg)	Cmax (μg/L)	Cmax/dose (μg/L/mg)	Tmax (min)	T½ (min)	AUClast (min*μg/L)	AUClast/dose (min*μg/L/mg)	AUCinf (min*μg/L)	AUCinf/dose (min*μg/L/mg)	AUC extrap (%)	Vz/F (L)	CL/F (L/min)
**1**	1.33	3.24	2.44	119	-	-	-	-	-	-	-	-
**2**	0.67	1.43	2.15	125	180	474	713	516	776	8	336	1.29
**3**	0.67	1.51	2.27	48	131	245	369	336	505	27	374	1.98
**4**	0.44	0.97	2.19	40	260	306	692	415	938	26	401	1.07
**5**	0.44	1.47	3.32	35	87	187	422	201	453	7	277	2.21
**6**	0.44	1.50	3.39	15	180	196	443	264	595	26	436	1.68
**7**	0.44	1.58	3.57	14	143	235	531	285	643	17	322	1.56

*ID*, identification number of the patient; *HM*, hydromorphone (free base); *Cmax*, peak plasma concentration; *Tmax*, time to maximum concentration; *T½*, half-life; AUClast, area under the plasma concentration-time curve from time zero to the last quantifiable concentration; *AUCinf*, area under the plasma concentration-time curve from time zero to infinity; *AUCextrap*, area under the plasma concentration-time curve extrapolated from the last quantifiable concentration to infinity as a percentage of total AUC; *Vz/F*, the apparent volume of distribution during terminal phase; *CL/F*, the apparent total body clearance

Dose-normalized results are presented for Cmax, AUClast and AUCinf. The results are calculated using hydromorphone free base

Epidural HM was given a median of 10 min (range 5–18) after the 10 mL test dose of lidocaine 5 mg/mL with adrenaline 10 μg/mL. All patients received additional levobupivacaine doses. The first dose was requested at a median of 163 min (range 19–303) after HM administration. Levobupivacaine was given on patient’s request. Contraction pain ratings and doses of levobupivacaine given are presented in Fig. 1. Three patients received only one subsequent dose of levobupivacaine, even though each of them had been administered a different dose of HM. Two parturients had two doses of levobupivacaine, and they had received the HM 0.5 mg. One participant required four doses of levobupivacaine over time period of 409 min and one had five doses over 730 min. Two doses of levobupivacaine were given prior to the HM onset of action, at 19 min after HM. The duration of delivery after receiving HM was a median of 385 min (range 229–552) in the six patients who delivered vaginally.

Twelve adverse effects were reported by seven parturients. The most common adverse effects were nausea (*n* = 5), vomiting (*n* = 3), pruritus (*n* = 3) and hypotension (*n* = 1). Patients ID 5 and ID 7 (both had HM 0.44 mg) suffered prolonged pruritus, which began from 3.6 to 4.3 h after receiving HM. Patient ID 1 (HM1.33 mg) suffered protracted nausea that began after delivery and was clearly an adverse effect of HM. Patients ID 2 (HM 0.67 mg), 3 (HM 0.67 mg), 5 (HM 0.44 mg) and 6 (HM 0.44 mg) had nausea prior to HM dosing, as well as briefly after HM administration.. Patient ID 4 (HM 0.44 mg) vomited ones 440 min after receiving HM without having any nausea and had mild pruritus. Hypotension, with a systolic pressure of 87 mmHg was treated with 3 mg boluses of ephedrine. None of the parturients experienced respiratory depression, and no supplemental oxygen was needed.

Our results suggest that HM crosses the placenta into the foetus as has been shown to other opioids [20]. Apgar and NAC scores of the newborns and HM concentrations in the umbilical artery and vein are presented in Table 3. A foetal/maternal-ratio (F/M-ratio) for HM concentrations was calculated from four parturients whose HM concentrations were above the lower limit of quantification. One newborn had an umbilical venous-HM concentration of 0.11 μg/L and in the mother’s plasma < 0.1 μg/L, i.e. F/M ratio would have been > 1.1. In six out of seven cases, the NACS was performed 30 min after birth. In one newborn, NACS was 30 and an Apgar score of 7/9/9. This newborn (ID 6) was born utilizing a vacuum extraction, remained under supervision of midwives, and further recovery of the baby was uneventful. None of the newborns required paediatric interventions.

**Table 3:** Maternal hydromorphone concentrations at the time of delivery, umbilical concentrations and newborn outcomes

ID	HM dose (mg) free base	HM free base dose/kg (μg/kg)	Maternal HM concentration at the time of delivery (μg/L)	Ua HM concentration (μg/L)	Uv HM concentration (μg/L)	F/M Ua	F/M Uv	Apgar	NACS
1	1.33	21	2.34	1.61	1.86	0.69	0.79	9,9,9	35
2	0.67	7	-	-	-	-	-	9,9,9	-
3	0.67	8	0.48	0.59	0.61	1.23	1,27	9,9,9	35
4	0.44	6.4	0.29	0.3	0.32	1.03	1.1	9,10,10	37
5	0.44	5.2	0.11	-	0.17	-	1.55	9,9,9	39
6	0.44	6.5	< 0.1	< 0.1	0.11	na	na	7,9,9	30
7	0.44	5.4	< 0.1	< 0.1	< 0.1	na	na	9,10,10	35

*ID*, identification number of the patient; *HM*, hydromorphone; *Ua*, uterine arterial; *Uv*, uterine venous; *F/M*, feto-maternal ratio; *na*, not applicable NACS, Neonatal neurological and adaptive capacity score

## Discussion

In the present study of labouring women, after epidural HM administration the median elimination half-life of 162 min (range 87–260) was observed, which is similar to that reported in non-pregnant adults receiving i.v. HM (mean 184 min) [19]. The distribution and elimination of epidural HM appeared to be fairly similar to that reported after i.v. administration [19] as the simulated concentration curve of epidural HM (bioavailability 100%) with distribution and elimination kinetics from i.v. study seemed parallel to the measured concentration curves. The three patients with the highest dosages (1.33 or 0.67 mg of HM free base) had higher Tmax values than the four patients receiving the lower dose (0.44 mg of HM free base). A more thorough pharmacokinetic study would be required to evaluate whether this observation was caused by random variability or by true biological or formulation-related phenomena. In the present study, blood sampling was discontinued as the baby was born, and the true elimination phase was not adequately captured for all parturients. For this reason, the reported pharmacokinetic parameters calculated based on the true elimination phase are approximate values and further research is needed to confirm the accuracy of these findings.

In the present study, 0.5 mg of HM hydrochloride resulted in Cmax range of 0.97–1.58 μg/L. As three different doses were administered, we used dose-normalization for parameter comparison. If HM follows linear kinetics, the pharmacokinetic parameters should increase proportionally as the dose is increased, and the dose-normalized parameters should be essentially the same. When dose-normalized results for Cmax were adjusted for the lowest dose (0.44 mg of HM free base), the median Cmax for the parturients was 1.08 μg/L (range 0.95–1.58 μg/L). Analgesic concentration of intravenous HM has been evaluated to our knowledge in two studies. In children with mucositis HM analgesic concentration was 4.7 μg/L [21] and in postoperative setting mean analgesic serum concentration was 4.1 μg/L [22].

Although Cmax values were lower in the present study than earlier reported, good pain relief was achieved with combination of local anaesthetic and HM. Plasma analgesic concentrations of HM after intravenous dosing are not comparable with plasma concentrations after epidural dosing as plasma concentration of HM depends on the rate of leaking from the epidural environment, as well as from the clearance of HM from the plasma, of which both are variable. Despite the low Cmax after epidural dosing, this could be perceived as evidence of HM’s spinal site of action when given epidurally.

Epidural HM pharmacokinetics has been studied in 16 thoracotomy patients [23]. In a study by Brose and co-workers, 0.75 mg HM was co-administered with 5 mg of morphine to a lumbar epidural catheter for postoperative pain relief. The mean HM Cmax of 14 μg/L was observed after 8 min and plasma HM concentrations remained at 5 μg/L or higher after 4 h [23]. These concentrations are much higher than in the present study despite similar dosing in two parturients. This might be an effect of the epinephrine administered in our test dose, which was administered 5–18 min prior to the HM and could slow HM migration into the systemic circulation [24]. In addition, the larger circulating plasma volume in pregnant than in non-pregnant patients may result changes in HM distribution. The glucuronidation of HM in pregnancy should be similar than in non-pregnant patients as UGT2B7 activity is unaltered during pregnancy based on zidovudine and morphine pharmacokinetic data [25]. Renal elimination of hydromorphone-3- glucuronide should be increased as maternal glomerular filtration rate increases during pregnancy. However low formation of metabolite of morphine by UGT2B7, morphine-3-glucuronide in neonates during their first days of life, has been reported [15]. This suggests immature glucuronidation of HM in the neonate that have to be considered when hydromorphone in labour analgesia is used. However, glomerular filtration rate in the neonates increases approximately 15% at birth and this increases slightly elimination of morphine and paracetamol [26], but we did not find data about hydromorphone elimination in newborns.

The adverse events in the present study were common to spinal opioids [5]. No severe adverse events occurred, none of the parturients in the present study had peripheral oxygen saturation less than 97% and none of them needed supplemental oxygen during the 12 h of observation. Lipophilic intrathecal sufentanil has been demonstrated to cause respiratory depression within 20 min due to its rapid rostral spread [3, 27]. We measured neither carbon dioxide concentrations nor respiratory rate, which might have been more sensitive methods than peripheral oxygen saturation [28].

The newborns had good Apgar scores. In five newborns, NACS was 35 or higher, but one had a NACS of 30 at 180 min after birth. The recovery of this newborn was uneventful. Foetal/maternal HM-ratio was less than unity in one case, ratio 0.8, and higher than 1.1 in three cases, suggesting that HM may accumulate into the foetus. However, differences in the pattern of clearance from the maternal circulation, transfer across the placenta, and clearance within the foetus, the ratio can vary widely depending on the time interval at which the last dose was administered to the mother. The ratio depends on the clearance capacity of the foetus which is low due to immature glucuronidation function in the liver, the rate of transport from foetal to maternal side over the placenta and the clearance rate of mother. The placental transport of drugs is affected by protein binding and ionization of the drug, free, non-ionized drug is transferred easily through placenta. As foetus is slightly acidic compared with mother ionization of drug may cause higher foetal concentrations of HM and may be reason to F/M ratio higher than 1 in the present study. However, F/M ratio could only be calculated in four out of seven parturients and this observation warrants further evaluation. Physiologically based pharmacokinetic (PBPK) modelling is increasingly used in pregnant patients due to certain limitations of traditional pharmacokinetic analysis. Paracetamol, another UGT substrate, has been evaluated with PBPK modelling and the developed pregnancy PBPK models successfully predicted pharmacokinetics of paracetamol and its metabolites at different stages of pregnancy [29]. Small cohorts as in the present study may benefit from PBPK modelling.

The main limitation of the present study was the small sample size. The number of parturients was limited to seven due to challenges in recruiting patients that fulfilled the inclusion criteria. Many parturients had received some other systemic opioid at the start of their labour and were therefore no longer eligible for recruitment to this study. Another limitation of the study was that the dose of HM was decreased from 1.5 to 0.75 and then to 0.5 mg due to nausea and vomiting induced by the higher doses. Due to changes in dose-levels, pharmacokinetic parameters were calculated dose-normalized. The kel and half-life were evaluated for 6 of 7 patients. The last data point available was obtained at the time of birth, and this resulted in a fairly short time interval in the terminal elimination phase in several subjects. In subjects 3, 4 and 6, the time interval was 1.5–1.8-fold compared with the estimated half-life. In the remaining three subjects, the ratio of the corresponding time interval to half-life was 2.3–3.3.

To conclude, in this pilot study in parturients, distribution and elimination of epidural HM seemed fairly similar to those observed earlier for non-pregnant participants receiving i.v. HM, when absolute bioavailability is assumed to be 100% for epidural HM. However, as one of the limitations in the study was the short sampling period in the terminal elimination phase, more research is needed to confirm pharmacokinetics of epidural HM in labouring women. Our results suggest that HM crosses the placenta like other opioids. The adverse events of HM were similar to those reported for spinal opioids. Doses lower than 0.5 mg should be used for single epidural dosing of HM hydrochloride to avoid adverse effects. We suggest maximal cumulative dose of 0.5 mg for the duration of the delivery in combination with local anaesthetics.
